# A pooled analysis of the LAMP assay for the detection of *Neisseria meningitidis*

**DOI:** 10.1186/s12879-020-05250-w

**Published:** 2020-07-20

**Authors:** Shu-Jin Fan, Hong-Kun Tan, Yu-Cheng Xu, Yuan-Zhi Chen, Tian-Ao Xie, Zhi-Yong Pan, Shi-Ou Yang, Qin Li, Xiao-yan Li, Zhen-Xing Li, Xu-Guang Guo

**Affiliations:** 1 Department of Clinical Laboratory Medicine, the Third Affiliated Hospital of Guangzhou Medical University, Guangzhou, 510150 China; 2 Department of Clinical Medicine, the Third Clinical School of Guangzhou Medical University, Guangzhou, 511436 China; 3 Department of infectious disease, The Fifth Affiliated Hospital of Guangzhou Medical University, Guangzhou, China; 4 Pulmonary, Critical Care and Sleep Medicine, Yale School of Medicine, New Haven, USA; 5 Department of Laboratory Medicine, The Affiliated Shunde Hospital of Guangzhou Medical University, Foshan, China; 6 Department of respiratory, The third Affiliated Hospital of Guangzhou Medical University, Guangzhou, China; 7 Key Laboratory for Major Obstetric Diseases of Guangdong Province, Guangzhou, 510150 China; 8 Key Laboratory of Reproduction and Genetics of Guangdong Higher Education Institutes, Guangzhou, 510150 China

**Keywords:** *Neisseria meningitidis*, Meningitis, LAMP assay, Pooled-analysis

## Abstract

**Background:**

*Neisseria meningitidis* is a major cause of bacterial meningitis, and these infections are associated with a high mortality rate. Rapid and reliable diagnosis of bacterial meningitis is critical in clinical practice. However, this disease often occurs in economically depressed areas, so an inexpensive, easy to use, and accurate technology is needed. We performed a pooled-analysis to assess the potential of the recently developed loop-mediated isothermal amplification (LAMP) assay for detection of meningococcus.

**Methods:**

Pubmed, Embase, and Web of Science were searched to identify original studies that used the LAMP assay to detect meningococcus. After pooling of data, the sensitivity and specificity were calculated, a summary receiver operating characteristic (SROC) curve was determined, and the area under the SROC curve was computed to determine diagnostic accuracy. Publication bias was assessed using Deek’s funnel plot.

**Results:**

We examined 14 studies within 6 publications. The LAMP assay had high sensitivity (94%) and specificity (100%) in the detection of meningococcus in all studies. The area under the SROC curve (0.980) indicated high overall accuracy of the LAMP assay. There was no evidence of publication bias.

**Discussion:**

The LAMP assay has accuracy comparable to bacterial culture and PCR for detection of meningococcus, but is less expensive and easier to use. We suggest the adoption of the LAMP assay to detect meningococcus, especially in economically depressed areas.

## Background

*Neisseria meningitidis* is a Gram-negative diplococci bacterium that only infects humans, and is a significant cause of meningitis. *Neisseria meningitidis* parasitizes in patient’s cerebrospinal fluid easily in the acute and early stage of diseases. But it is unknown of the carriage rate in healthy individuals at presen t[1]. Based on capsular polysaccharides, there are 13 serogroups of this species (A, B, C, D, E, H, I, K, L, W, X, Y, and Z )[2] Serogroups A, B, C, Y, and W-135 are mainly responsible for human diseases,[3] and serogroups A and C are mainly responsible for meningitis. By the way we found other groups such as group B, Y and W135 cause severe epidemic lots of lands. The incidence of bacterial meningitis is greatest among infants under 1 year-old, followed by adolescent s[4].

The overall incidence of meningitis has declined in recent decades due to the increasing use of meningococcal vaccines,[5] but the incidence of meningitis from bacterial strains not covered by vaccines has increased, and bacterial meningitis remains a significant public health problem. About 200,000 patients worldwide die from bacterial meningitis every year, and the mortality can be up to 60% in parts of sub-Saharan Africa and in poor and developing countrie s[6, 7]. Patients who receive treatment have a mortality rate of about 10%, but survivors often experience serious sequelae, including limb amputation, neurological deficits, and other serious disabilitie s[8].

Clinical detection of *Neisseria meningitidis* in many countries currently uses PCR, ELISA, and bacterial culture,[9] and PCR is considered the most authoritative standard. However, these technologies have limited applicability in poor and developing countries. For example, PCR and ELISA can be time-consuming, difficult to perform, and relatively expensiv e[10]. Identification by bacterial culture can take many days, has low sensitivity, and has a reduced detection rate for patients who were pretreated with antibiotic s[6].

Loop-mediated isothermal amplification (LAMP) is a relatively new nucleic acid amplification technology that can simultaneously detect six meningococcal groups of *N. meningitidis* (A, B, C, W, X, and Y). It is simple to perform, rapid, has high sensitivity, and is inexpensive,[11] making it especially suitable for developing countries and regions with limited resources. No previous studies have comprehensively analyzed the sensitivity and specificity of the LAMP assay for detection of *Neisseria meningitidis*. The present analysis evaluated the diagnostic accuracy and feasibility of using LAMP to identify meningitis due to *Neisseria meningitidis*. This analysis also included the assessment of publication bias and data quality.

## Methods

### Study design

A systematic review of the diagnostic accuracy of LAMP in *Neisseria meningitidis* was performed, followed by a pooled-analysis.

#### Search strategy and study selection

The phrases “Neisseria meningitidis”, “Meningococcus”, “LAMP” and “Loop-mediated isothermal amplification” were used in combination to systemically search the literature from January 1997 to February 7, 2019 in 5 databases (Pubmed, Embase, Medline, Web of Science, and Cochrane Library) for identification of original studies that used LAMP to detect *Neisseria meningitidis*. The bibliographies of all publications were also reviewed to identify additional studies.

All literature titles and abstracts were independently screened, and five researchers (including Fan Shujin and Tan Hongkun) read the full text of each article that had extractable data to determine its eligibility for inclusion. Differences regarding eligibility were resolved through negotiation. Each included study used LAMP for detection of *Neisseria meningitidis* and a gold standard test (PCR or culture); provided data that were extractable and included true positivity (TP), false positivity (FP), false negativity (FN), and true negativity (TN) of the LAMP assay; and was original research written in English or Chinese. Studies that were animal experiments, from conferences or literature reviews, or did not have extractable data were excluded.

### Data extraction and quality assessment

Five researchers independently extracted the data from each included study, including author, publication year, sample size, sample type, gold standard test results, TP, FP, FN, and TN. Regarding patient selection, index test, reference standard, flow and timing were used to evaluate the diagnostic accuracy of the LAMP assay.

### Statistical analysis

MetaDisc version 1.4.0.0 was used to calculate sensitivity, specificity, positive likelihood ratio (PLR), negative likelihood ratio (NLR), and diagnostic odds ratio (DOR). Diagnostic odds ratio was used as the main outcome measure of the SROC approach of the standard method for analyzing diagnostic studies reporting pairs of sensitivity and specificity. A summary receiver operating characteristic (SROC) curve was plotted for calculations of sensitivity and specificity. A random effects model was used to summarize all data. Deek’s funnel plot was drawn using STATA, and a bivariate box plot and a post-test probability plot were used to detect literature bias. Review Manager version 5.2 was used for plotting study quality evaluation.

## Results

### Search results and study characteristics

Our initial literature search identified 83 publications and no additional (gray) literature (Fig. S1). After application of the inclusion and exclusion criteria, we identified 6 publications for inclusion in the analysis, all of which were published between 2011 and 2018 (Table 1). These studies were performed in Ireland, the United Kingdom, South Korea, and Japan. Because some of these publications used multiple types of sampling, we categorized these 6 publications as 14 studies with 3185 samples.

**Table 1:** Characteristics of the included studies

Author	Year	Country	Patients (n)	Specimen	Referencestandard	TP	FP	FN	TN
McKenna et al .[12]	2011	UK	139	Throat swab	PCR	12	1	0	126
McKenna et al .[12]	2011	UK	141	Serum	PCR	14	1	0	126
McKenna et al .[12]	2011	UK	72	Blood	PCR	4	1	0	67
McKenna et al .[12]	2011	UK	22	CSF	PCR	3	1	0	18
McKenna et al .[12]	2011	UK	16	Respiratory secretions	PCR	2	0	0	14
McKenna et al .[12]	2011	UK	4	Feces	PCR	1	0	0	3
Bourke et al .[13]	2015	U.K.	141	Nasopharyngeal	PCR	21	0	4	116
Bourke et al .[13]	2015	U.K.	144	Blood	PCR	22	0	4	118
Lee et al .[14]	2015	South Korea	31	CSF	Culture	2	3	0	26
Lee et al .[15]	2015	South Korea	568	CSF	PCR	15	5	0	548
Lee et al .[15]	2015	South Korea	536	CSF	PCR	5	0	0	531
Lee et al .[15]	2015	South Korea	470	CSF	PCR	5	1	0	464
Takahashi et al .[16]	2016	Japan	836	Throat swab	PCR	6	1	0	829
Higgins et al .[17]	2018	UK	65	Blood	PCR	22	0	0	43

*CSF* cerebrospinal fluid, *FN* false negative, *FP* false positive, *TN* true negative, *TP* true positive

### Diagnostic accuracy of LAMP assay

**Fig. 1:**
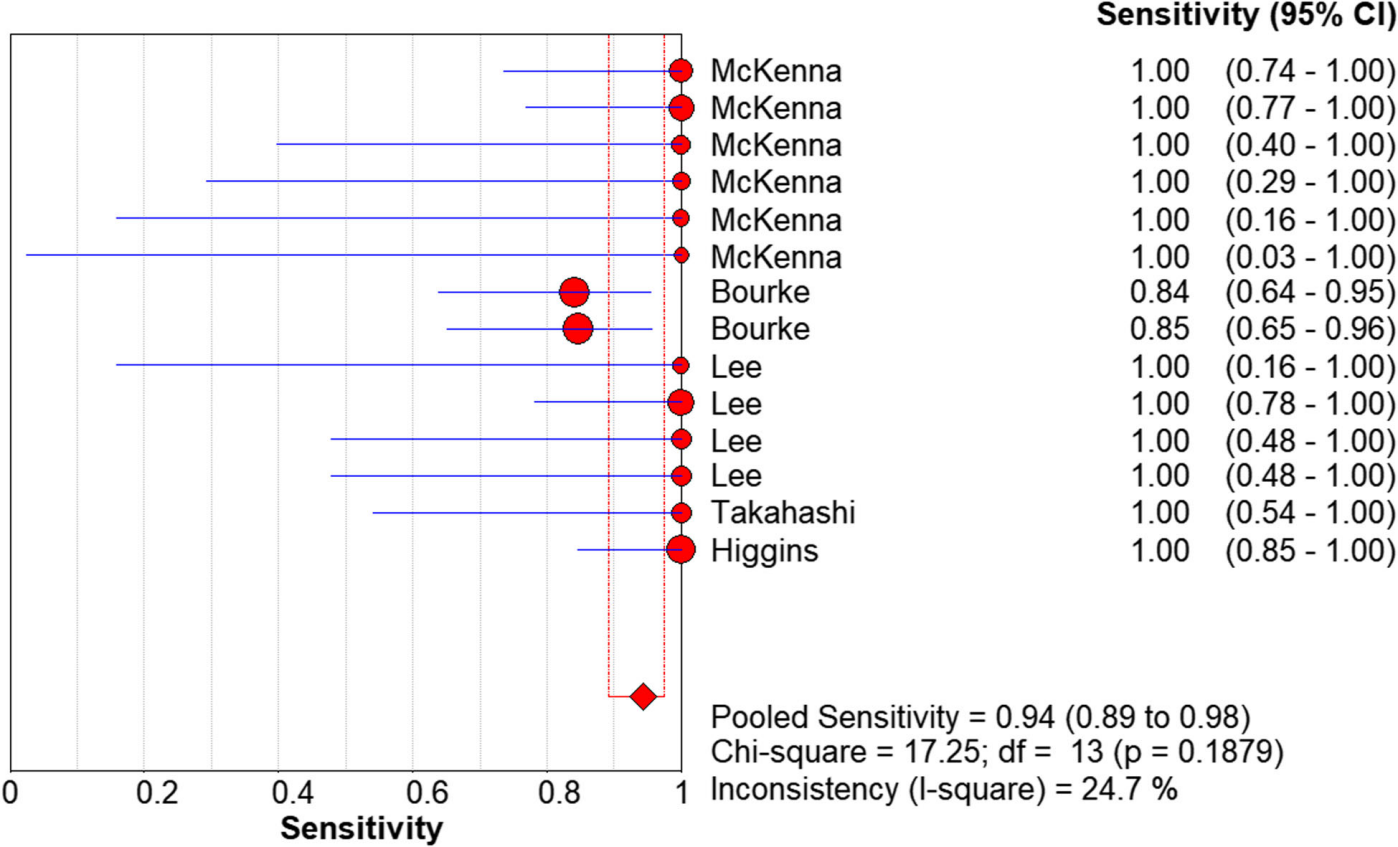
Sensitivity of the included studies

**Fig. 2:**
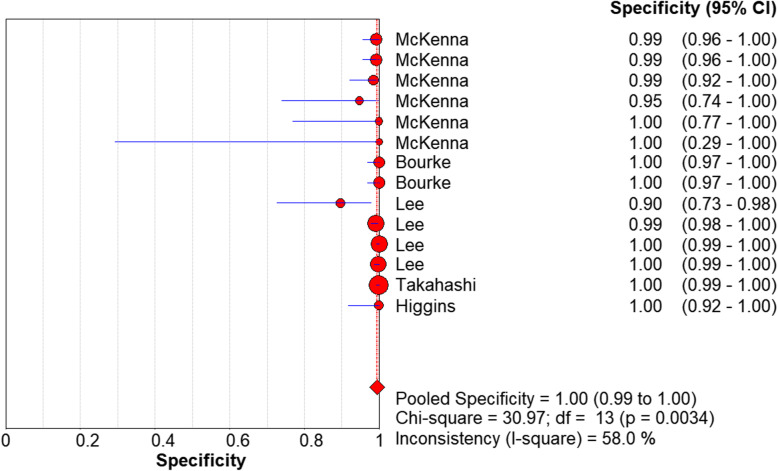
Specificity of the included studies

**Fig. 3:**
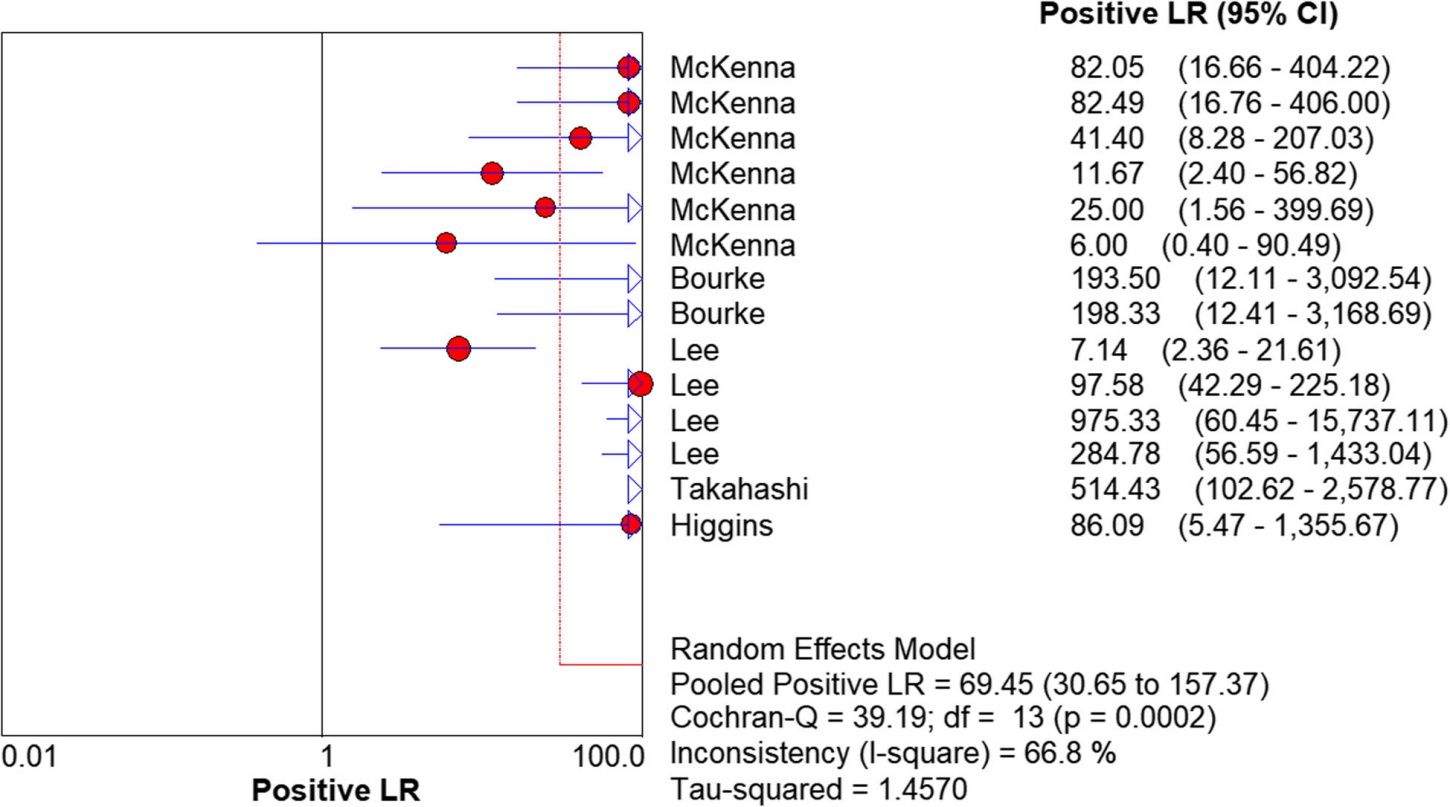
Positive LR of the included studies

**Fig. 4:**
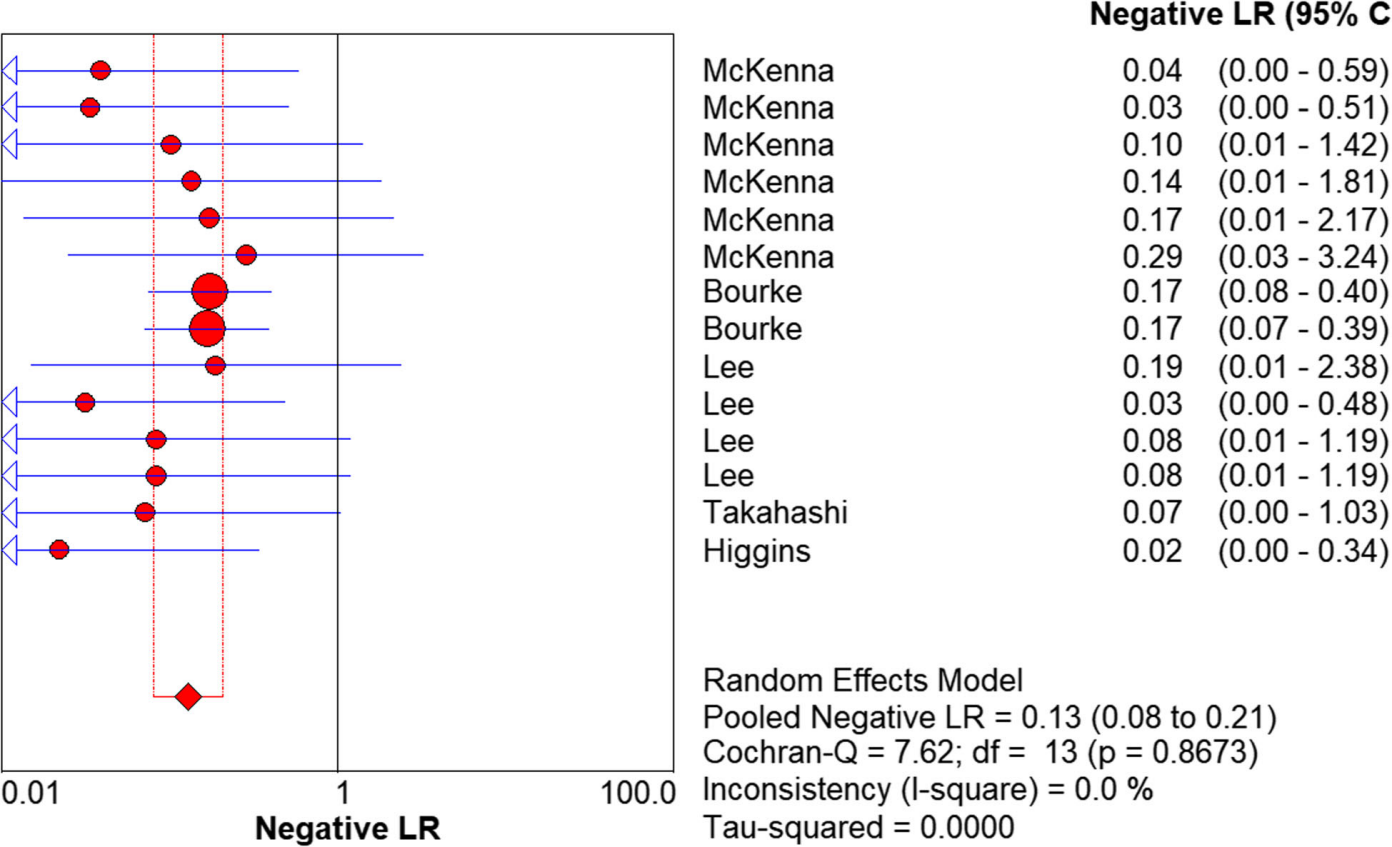
Negative LR of the included studies

**Fig. 5:**
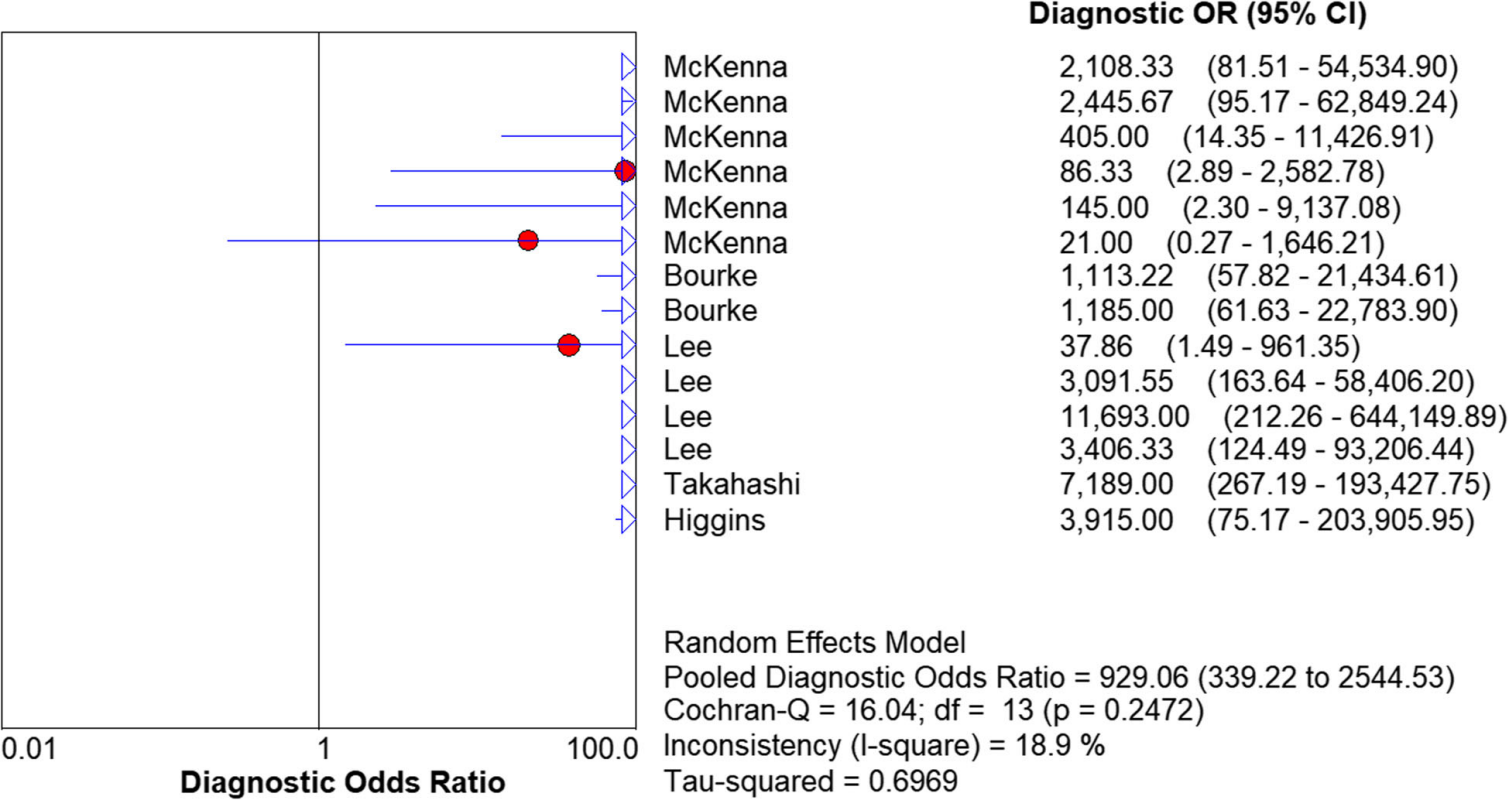
Diagnostic OR of the included studies

**Fig. 6:**
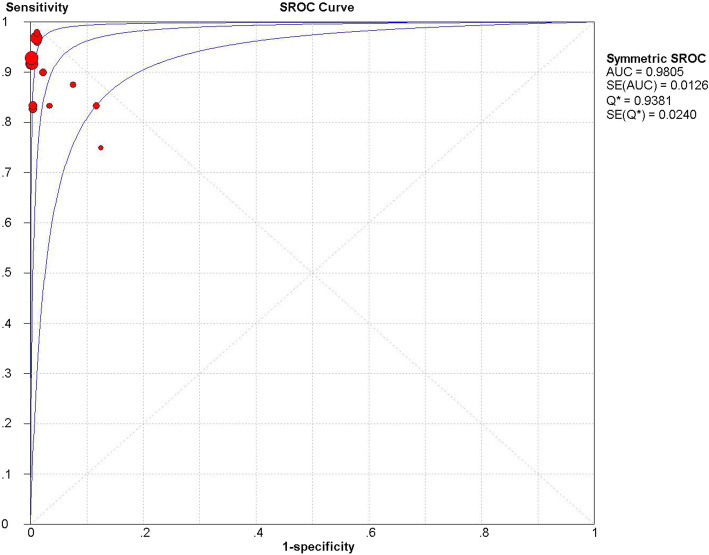
SROC curve of LAMP for diagnosis of *N. meningitidis*

We used a random effects model to determine the diagnostic accuracy of the LAMP assay. Overall, the sensitivity was 0.94 (95% CI = 0.89 to 0.98, I^2^ = 24.7%; Fig. 1), the specificity was 1.00 (95% CI = 0.99 to 1.00, I^2^ = 58%; Fig. 2), the PLR was 69.45 (95% CI = 30.65 to 157.37, I^2^ = 66.8%; Fig. 3), the NLR was 0.13 (95% CI = 0.08 to 0.21, and I^2^ = 0.0%; Fig. 4), the DOR was 929.06 (95% CI = 339.22 to 2544.53, I^2^ = 18.9%; Fig. 5), and SROC analysis indicated the Area Under Curve (AUC) was 0.9805 (Fig. 6).

### Quality evaluation

**Fig. 7:**
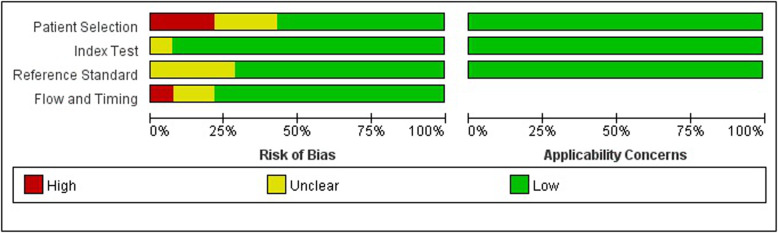
Overall quality assessment of the included studies

**Fig. 8:**
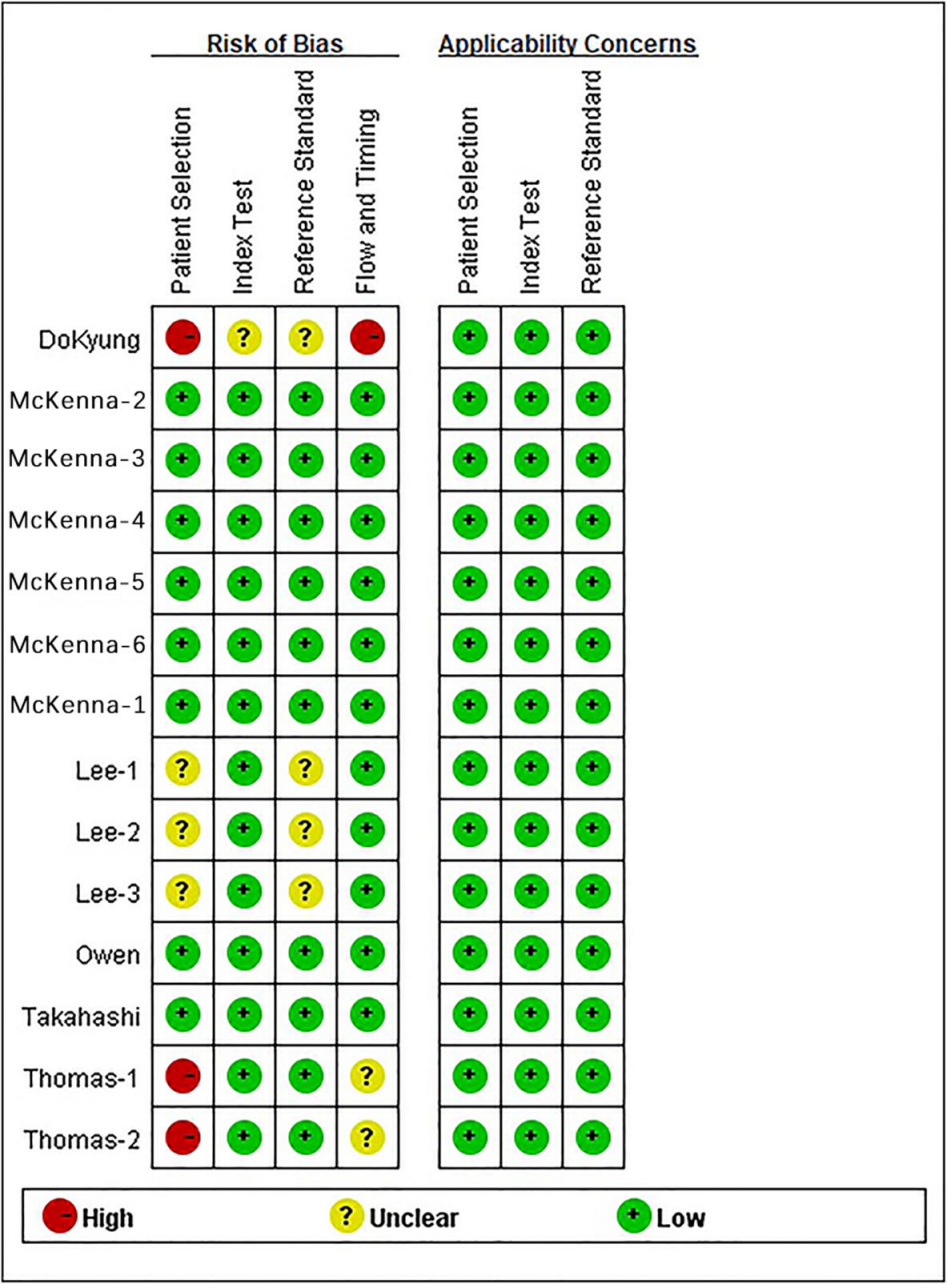
Quality assessment of the individual studies

We assessed the quality of the studies by analysis of patient selection, the index test, the reference standard, and study flow and timing (Figs. 7 and 8). The results indicated that patient selection had a high risk of bias in 3 studies, an unclear risk of bias in 3 studies, and a low risk of bias in 8 studies. Analysis of patient flow and timing indicated that only 1 study had a high risk of bias and 2 studies had an unclear risk of bias. Analysis of the index test indicated an unclear risk of bias in 1 study, and low risk of bias in the other 13 studies. Analysis of reference standards indicated an uncertain risk of bias in 4 studies and low risk of bias in 9 studies. Analysis of applicability concerns indicated low concerns in all three categories.

### Publication bias

**Fig. 9:**
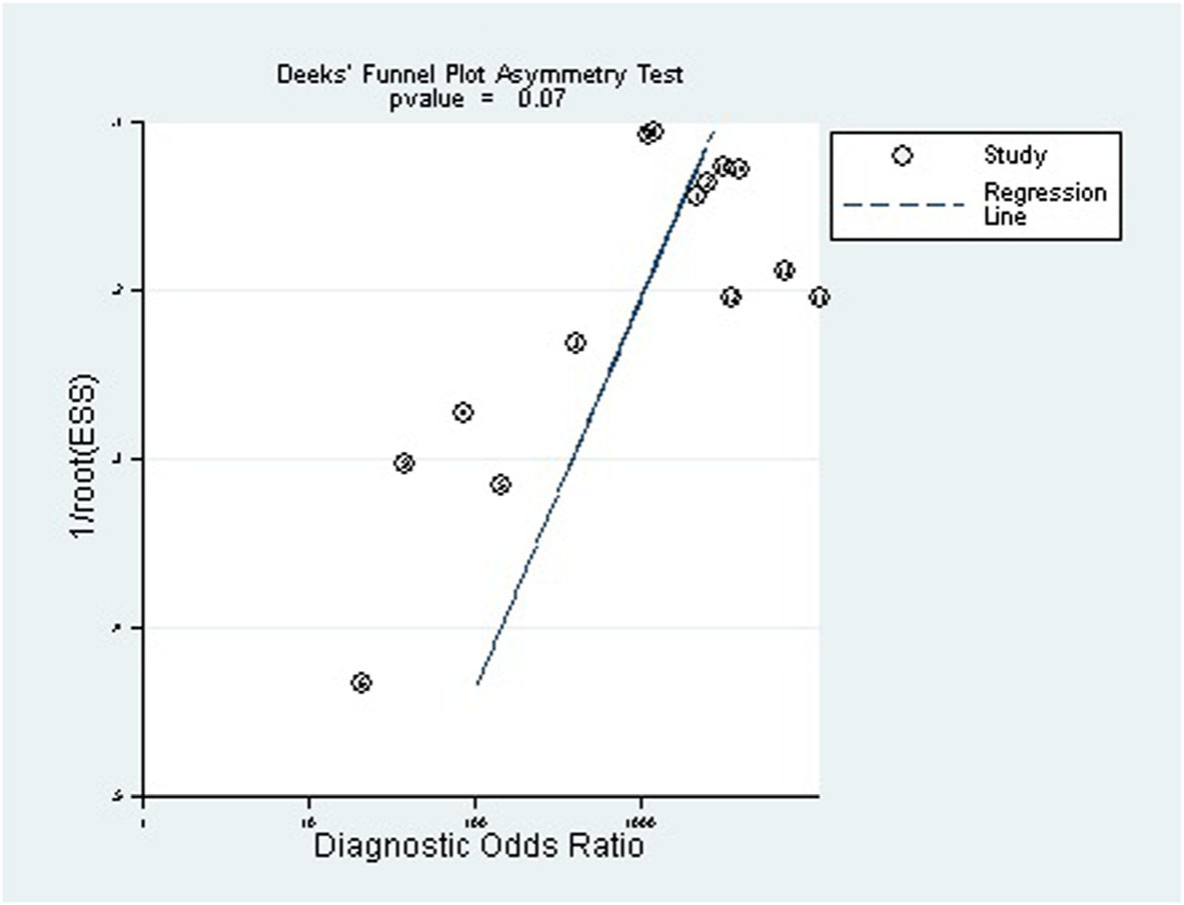
Deek’s funnel plot asymmetry test

**Fig. 10:**
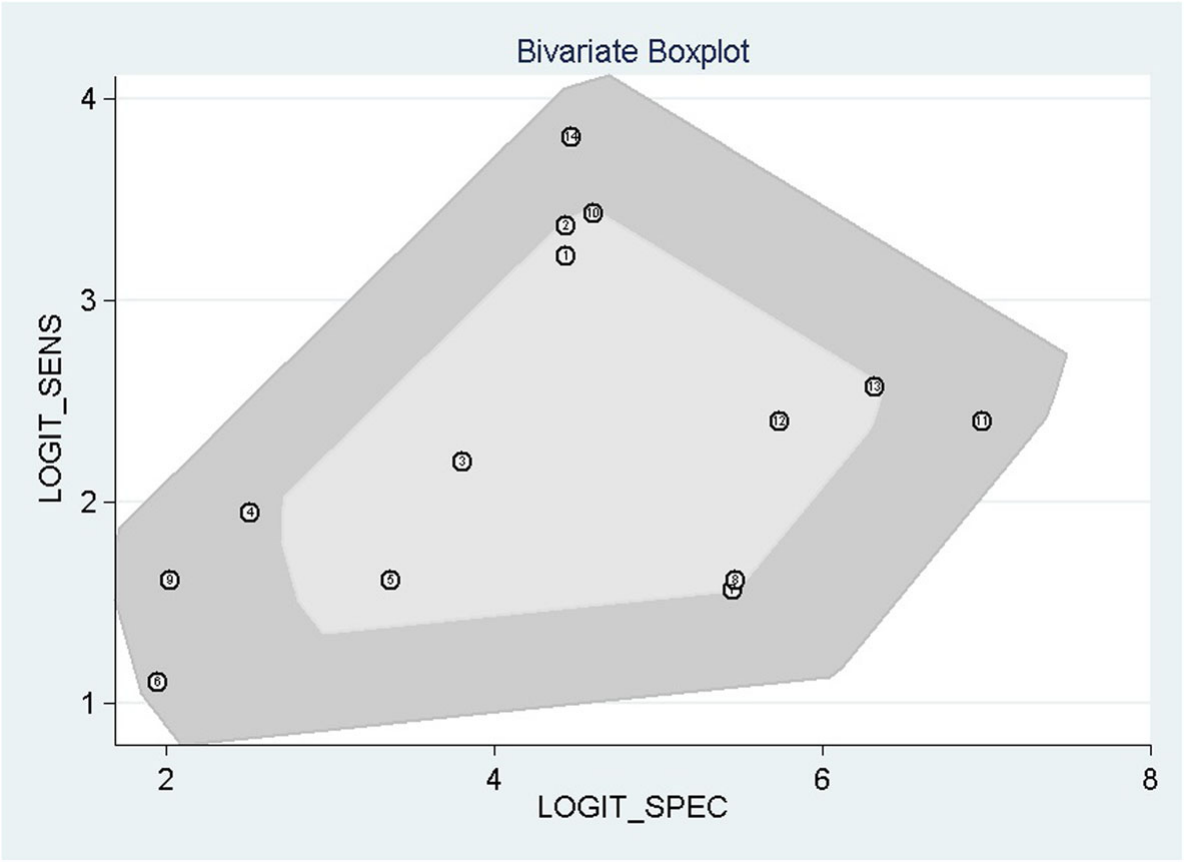
Bivariate boxplot of the relationship of sensitivity and specificity

**Fig. 11:**
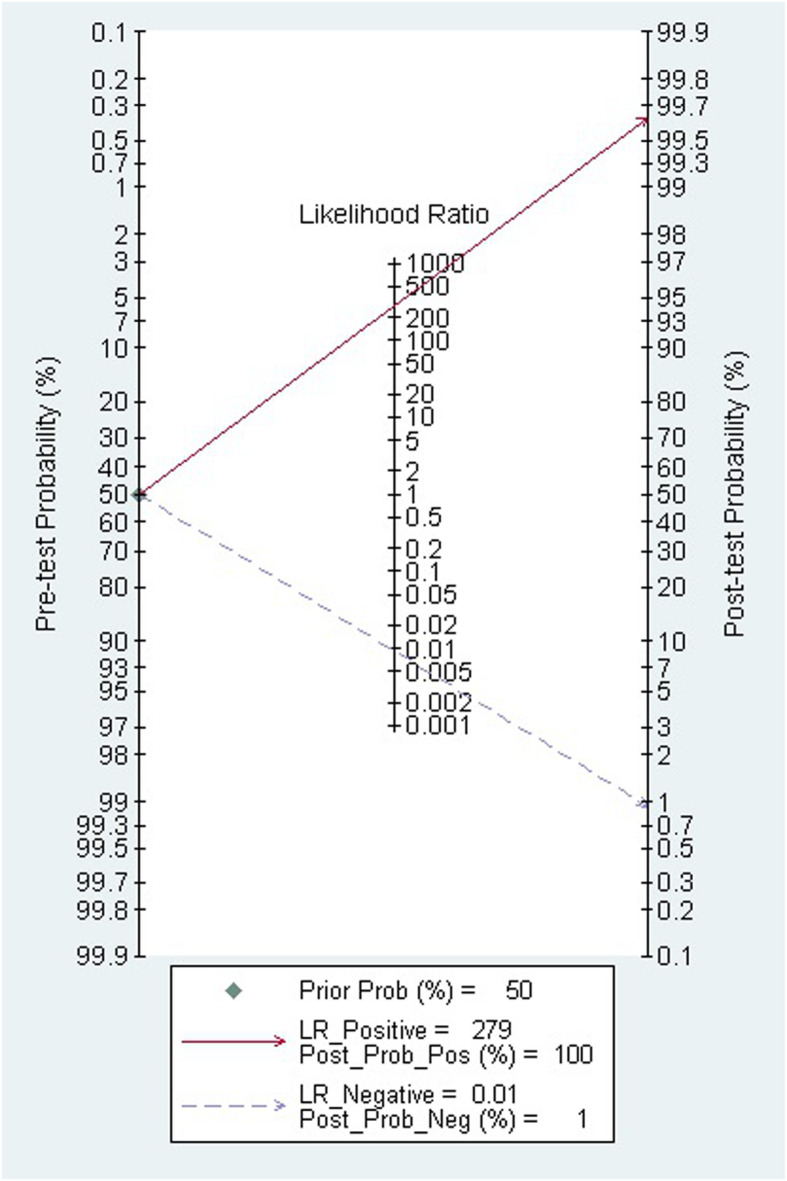
Fagan nomogram of disease probabilities based on Bayes’ theorem

Deek’s funnel plot (Fig. 9) showed that publication bias was not significant (*P* = 0.07). A bivariate box plot of sensitivity and specificity showed that there was no significant heterogeneity among the included studies (Fig. 10). The post-test probability nomogram showed that for samples with a predicted probability of 50%, the post-test probability of a positive result was 100%, and the post-test probability of a negative result was 1% (Fig. 11).

## Discussion

Bacterial meningitis caused by *Neisseria meningitidis* has high global incidence and mortality rates, and is an especially severe problem in undeveloped regions. At present, routine laboratory diagnosis of bacterial meningitis employs bacterial culture and Gram staining, but this method is limited because it has a low sensitivity and is very time-consumin g[18, 19]. PCR is a widely accepted laboratory test for diagnosis of bacterial meningitis, and many studies have confirmed its high sensitivity and specificity. Unfortunately, the current PCR applications used to detect bacterial meningitis are too complicated and expensive for clinicians in undeveloped region s[19, 20]. Thus, existing PCR tests are difficult to apply in areas with the highest incidences of bacterial meningitis. A rapid, low-cost, easy to use, and highly sensitive technique is needed for these at-risk populations.

LAMP is a simple technique developed in recent years that uses 4 specific primers in 6 regions of the target gene and does not require a thermocycler. Under the action of a strand displacement DNA polymerase (Bst DNA polymerase), genes are amplified at a constant temperature of 60 to 65 °C, and amplification can be 10^9^-fold to 10^10^-fold in 15 to 60 min. Higgins et al .[17] used *Tth* endonuclease IV and a unique LAMP primer/probe to develop novel real-time multiplex LAMP technology, TEC-LAMP. Its main advantages are that it is simple, fast, and inexpensiv e[12, 21].

We performed a pooled-analysis of 14 independent studies reported in 6 publications. These studies were performed in diverse geographic regions and used a variety of different samples. Thus, the study of McKenna et al .[13] was from Ireland and had 139 throat swab samples, 141 plasma samples, 72 blood samples, 22 cerebrospinal fluid samples, 16 respiratory secretion samples, and 4 stool samples. The study of Bourke et al .[14] was from the United Kingdom and had 141 nasopharyngeal swab samples and 144 blood samples. The study of Lee et al .[15] was from South Korea and examined 31 cerebrospinal fluid samples. The study of Lee et al .[16] was also from South Korea and examined 1574 cerebrospinal fluid samples (568 from Vietnam, 536 from China, and 470 from South Korea). The study of Takahashi et al .[1] was from Japan and examined 836 throat swab samples. The study of Higgins et al .[17] was from Ireland and examined 65 blood samples. The study of Lee et al .[14] used culturing as the reference standard, and all the other studies used PCR as the reference standard. Our analysis results indicated that LAMP had an overall sensitivity of 94% (95% CI = 0.89 to 0.98) and an overall specificity of 100% (95% CI = 0.99 to 1.00) for the detection of *N. meningitidis*, and that the accuracy was high regardless of the sample type and geographic origin of the patients. Therefore, LAMP is highly suitable for the rapid, accurate, and inexpensive clinical detection of *N. meningitidis* in economically impoverished areas. Further prospective studies are needed to verify the safety and most suitable samples for the clinical use of LAMP.

## Conclusions

In conclusion, our research showed that the LAMP assay had high sensitivity and specificity in detecting bacterial meningitis due to *N. meningitidis*. This assay thus provides reliable information for clinical laboratory tests, and is suitable for detecting bacterial meningitis. However, further research is needed to verify the clinical feasibility and accuracy of this test in the impoverished areas where it is most needed.

## Supplementary information

**Additional file 1: Figure S1.** Identification and selection of the included studies.

## Data Availability

All data generated or analyzed during this study are included in this published article and its supplementary information files.

## Abbreviations

LAMP: Loop-mediated isothermal amplification
SROC: Summary receiver operating characteristic
PCR: Polymerase Chain Reaction
ELISA: Enzyme Linked Immuno Sorbent Assay
TP: True positivity
FP: False positivity
FN: False negativity
TN: True negativity
PLR: Positive likelihood ratio
NLR: Negative likelihood ratio
DOR: Diagnostic odds ratio
