# Correction to: A pooled analysis of the LAMP assay for the detection of *Neisseria meningitidis*

**DOI:** 10.1186/s12879-020-05300-3

**Published:** 2020-07-31

**Authors:** Shu-Jin Fan, Hong-Kun Tan, Yu-Cheng Xu, Yuan-Zhi Chen, Tian-Ao Xie, Zhi-Yong Pan, Shi Ouyang, Qin Li, Xiao-yan Li, Zhen-Xing Li, Xu-Guang Guo

**Affiliations:** 1 Department of Clinical Laboratory Medicine, the Third Affiliated Hospital of Guangzhou Medical University, Guangzhou, 510150 China; 2 Department of Clinical Medicine, the Third Clinical School of Guangzhou Medical University, Guangzhou, 511436 China; 3 Department of infectious disease, The Fifth Affiliated Hospital of Guangzhou Medical University, Guangzhou, China; 4 Pulmonary, Critical Care and Sleep Medicine, Yale School of Medicine, New Haven, USA; 5 Department of Laboratory Medicine, The Affiliated Shunde Hospital of Guangzhou Medical University, Foshan, China; 6 Department of respiratory, The third Affiliated Hospital of Guangzhou Medical University, Guangzhou, China; 7 Key Laboratory for Major Obstetric Diseases of Guangdong Province, Guangzhou, 510150 China; 8 Key Laboratory of Reproduction and Genetics of Guangdong Higher Education Institutes, Guangzhou, 510150 China

**Correction to: BMC Infect Dis (2020) 20:525**

**https://doi.org/10.1186/s12879-020-05250-w**

Following publication of the original article [1], the authors identified an error in the author name of dr. Shi Ouyang.

The incorrect author name is: Shi-Ou Yang

The correct author name is: Shi Ouyang

The author group has been updated above and the original article [1] has been corrected.
