# Supplementary figures and images for: A pooled analysis of the LAMP assay for the detection of Neisseria meningitidis
Source: BMC Infect Dis. 2020 Jul 20;20:525. doi: 10.1186/s12879-020-05250-w (PMC7372874; doi:10.1186/s12879-020-05250-w)

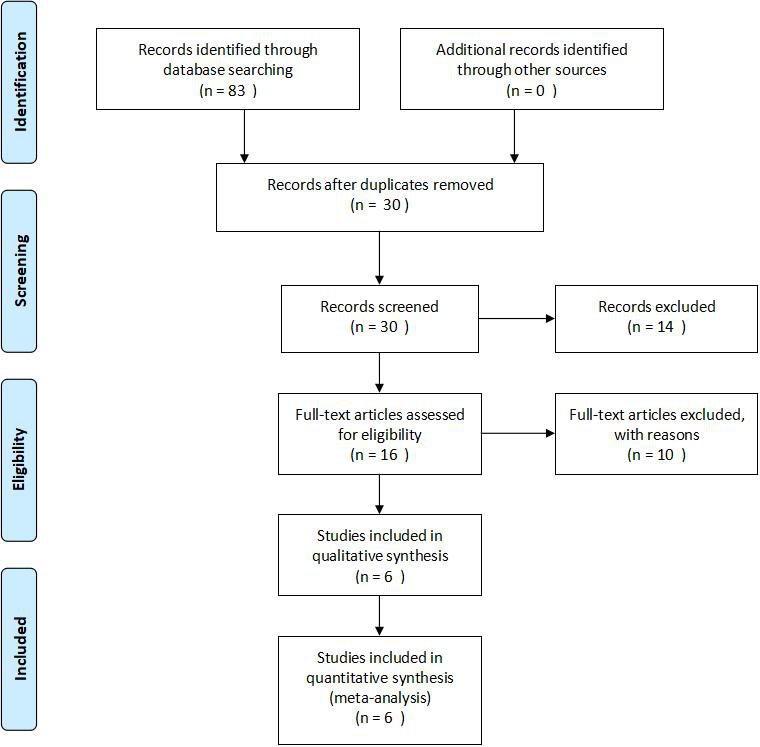

Supplement: Supplementary file 1 — Additional file 1: Figure S1. Identification and selection of the included studies. [file 12879_2020_5250_MOESM1_ESM.png]
